# Iatrogenic orbital hematoma: an exceptional complication of upper gastrointestinal endoscopy

**DOI:** 10.2144/fsoa-2023-0115

**Published:** 2023-09-08

**Authors:** Myriam Ayari, Sameh Riahi, Imen Abdelaali, Taieb Jomni, Mohamed Hedi Douggui

**Affiliations:** 1 Gastroenterology Department, Internal Security Forces Hospital La Marsa, Tunis, 2070, Tunisia; 2 University of Tunis El Manar, Tunis, 1068, Tunisia

**Keywords:** arterial hypertension, iatrogenic orbital hematoma, non-traumatic orbital hematoma, upper gastrointestinal endoscopy

## Abstract

Iatrogenic orbital hematoma is a rare event, most often manifested by a painful exophthalmos that could compromise the patient's visual prognosis. We report the case of a 51-year-old female patient with a history of non-ischemic dilated cardiomyopathy and high blood pressure, who developed a painful exophthalmos following an upper gastrointestinal sedation-free endoscopy. The diagnosis of an intra-orbital hematoma was made by computed tomographic scan and a conservative attitude was adopted after a thorough ophthalmological examination. Upper endoscopy may trigger this condition in the presence of pre-existing predisposing factors such as blood clotting disorders, high blood pressure or vascular malformation. This case highlights an uncommon complication of a commonly performed endoscopy that endoscopists should be aware of to provide safe and optimal examination.

## Plain language summary

A complication called iatrogenic orbital hematoma is a rare occurrence that can potentially harm a patient's vision. We report a 51-year-old patient who experienced a painful bulging of her eye after undergoing an upper endoscopy (a common procedure to look inside the upper digestive tract) without sedation. A CT scan helped doctors diagnose the problem and after a thorough examination, the condition resolved by itself. This case shows that upper endoscopy can lead to complications and endoscopists should be aware of this possibility to make sure the procedure is safe and successful.

Orbital hematoma is a rare condition outside the context of facial or orbital trauma. Early diagnosis enables appropriate management, preserving the ocular functional prognosis. It is often manifested by eye proptosis and ocular motor disorders with diagnostic confirmation made by computed tomography (CT) scan or magnetic resonance imaging. Orbital hematoma can be responsible of an increase in intra-ocular pressure, which leads ultimately to an ischemia of the optic nerve and the central retinal artery compromising the vision. Non-traumatic intra-orbital hematoma may complicate or reveal vascular malformations, hypertension, blood flow disorders such as sudden rise in venous pressure, and other causes of inflammatory, tumor or vascular orbital processes. Treatment options are ranging from observation to needle aspiration, and even surgery if the visual prognosis is threatened by a compressive optic neuropathy. Here in, we report an unusual case of iatrogenic orbital subperiosteal hemorrhage following sedation-free upper endoscopy as a reminder of the invasive character and therefore not totally harmless aspect of this examination.

## Case presentation

**Figure 1.**
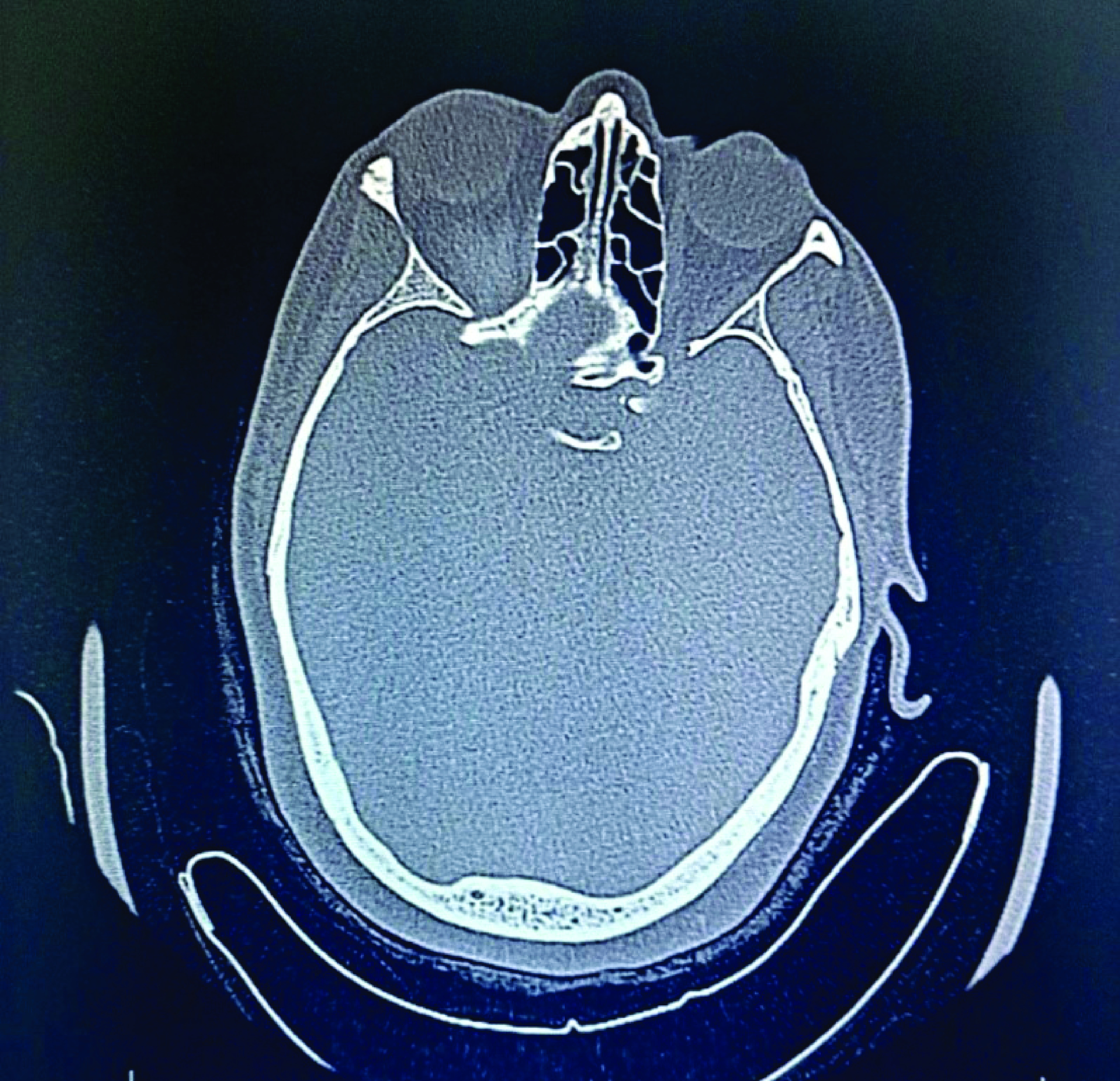
Axial computed tomography scan showing the hemorrhage of the right orbit.

**Figure 2.**
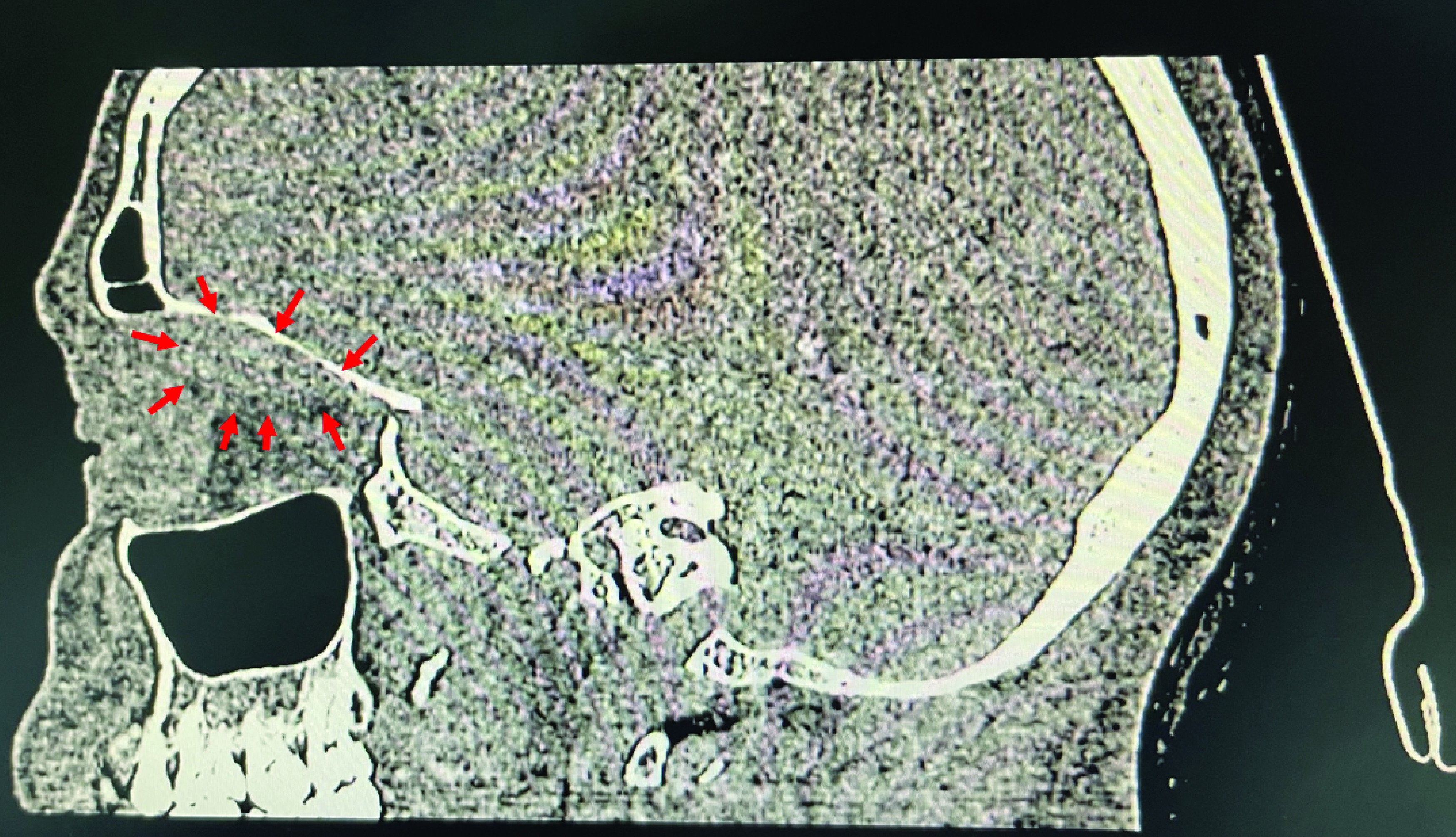
Sagittal computed tomography scan showing a demarcated soft tissue density lesion in the right superior subperiosteal area.

A 51-year-old Caucasian woman presented to the endoscopy unit for a scheduled gastroscopy without sedation. Past medical history included high blood pressure, non-ischemic dilated cardiomyopathy and dyslipidemia. The indication of the endoscopic examination was epigastralgia with iron deficiency anemia. The patient was not under anti-coagulant or anti-platelet therapy. An upper endoscopy procedure was performed, during which the patient experienced symptoms of nausea and excessive gagging. The examination revealed a congestive and nodular antropathy. Gastric and duodenal biopsies were performed. Ten min after the end of the examination, the patient reported a sudden onset of impulse and pain in her right globe which was displaced forwards. She also experienced light visual blur. Examination at the endoscopy room revealed palpebral swelling and proptosis. Specialized ophthalmic examination showed normal visual acuity (10/10), normal intraocular pressure and conserved ocular motor function on both sides. No abnormalities were detected upon examination of the cornea, the lens and the fundus. Initially, sinus fracture or intra-orbital hematoma was suspected. Therefore a CT scan without contrast injection of the sinus was performed for further investigation. Axial (Figure 1) and sagittal (Figure 2) views showed a thinned appearance of the cortical bone of the roof of the right orbit, in contact with a circumscribed formation, roughly oval, spontaneously hyperdense, measuring 27 × 10 mm, exerting a mass effect on the ipsilateral eyeball which is pushed back downwards and forwards responsible for a grade I exophthalmos. In addition, the exam showed normal pneumatization of the sinuses of the face, free ostiomeatic junctions, median nasal septum without bone spur, free nasal cavity and cavum. Thus, the CT concluded to a right orbital hematoma causing a grade I proptosis. Given that that were no signs of optic nerve compression, the patient was managed conservatively with spontaneous clinical resolution.

## Discussion

The most frequent cause of orbital hematoma are traumatic or related to sinonasal surgery [1]. Non-traumatic orbital hemorrhage (NTOH) is an uncommon condition affecting with predilection women of all ages. The causes are still not fully understood [2]. The clinical presentation is often brutal in the form of ocular pain, exophthalmia and edema. Visual acuity may be affected in variable degrees. Anatomic patterns of NTOH include diffuse intraorbital hemorrhage, ‘encysted’ hemorrhage (hematic cyst), subperiosteal hemorrhage, hemorrhage in relation to extra ocular muscles and hemorrhage related to orbital floor implants [3]. Diagnosis is often easy in the acute and subacute phase, based on imaging. A spontaneous hyperdensity of the lesion on a CT scan is strongly suggestive. Magnetic resonance imaging (MRI) shows a hypersignal on T1- sequences and a hyposignal on T2- sequences. Away from the acute phase, when the hematoma becomes liquefied, MRI shows hyposignal on both T1 and T2 sequences [4]. The presence of venous anomalies has been suggested as responsible of the occurrence of NTOH. However, anomalies of the arterial wall, such as an aneurysm of the ophthalmic artery, have also been noted in some patients with advanced age and pronounced atherosclerosis. The clinical presentation in these cases was more severe in terms of pain intensity and visual acuity impairment [2]. Orbital hematoma in the setting of diagnostic upper endoscopy is extremely rare. In the literature, only three cases were described. The first reported a case of NTSOH following upper gastrointestinal endoscopy in a patient with a history of viral C cirrhosis. The patient underwent subsequent endoscopic variceal ligation for grade III esophageal varices, and developed left sided preseptal and subperiosteal hemorrhage [5].

The second report detailed a 38-year-old patient who presented with an orbital hemorrhage two days after she had undergone an upper endoscopy indicated for hematemesis and done under local pharyngeal anesthesia, during which the patient had a few episodes of forceful retching [6]. More recently, Lotlikar *et al.* described the case of a 37-year-old woman followed for non-cirrhotic portal hypertension with pancytopenia and normal coagulation profile, presenting with painful bilateral eye swelling within 30 min after gastroscopy performed to investigate hematemesis. CT scan revealed bilateral organized hematoma which was managed conservatively by platelet transfusion and supportive ophthalmic care [7].

The occurrence of NTOH following upper endoscopy can be explained by a sudden increase in the intrathoracic and intra-abdominal pressures during retching, which cause rising of central venous pressure. The most likely mechanism for this condition is the transmission of the increased central venous pressure to the orbital vasculature, which is valveless. This hypothesis is further supported by the case of a 36-year-old man diagnosed with a subperiosteal orbital hematoma after thoracoabdominal crush injury without facial fracture and by the case of a 32-year-old woman in labor in which spontaneous bilateral orbital subperiosteal hemorrhage occurred [8,9]. An increase in intracranial venous pressure during the valsalva maneuver leading to subperiosteal orbital hematoma has also been noted [10]. However, it seems insufficient on its own to justify the occurrence of this event and appears rather as a triggering factor that is often associated with other underlying liver diseases as the cases reported above. The literature cites also, in this context, the spontaneous occurrence of an intra-orbital hematoma in a 60-year-old patient with advanced cirrhosis, during coughing fits [5] and in a 33-year-old women with acute liver failure [11]. Other situations were less frequently associated with this event, such as, blood diseases and arterial hypertension as in the case of our patient. In this late case, the intraorbital hematoma would then be the consequence of the rupture of a small vascular ectasia simultaneously to hypertensive peak [4] that may be induced during upper endoscopy.

## Conclusion

Although diagnostic upper endoscopy is usually a safe procedure, rare complications such as iatrogenic orbital hematoma can occur. Endoscopists should be aware of this potential uncommon event of a commonly performed procedure to both prevent its occurrence and optimize the management in order to minimize downstream harm once happened. Therefore, care should be taken to avoid retching during the procedure by checking for the gag reflex before endoscopy for a better selection of the patients undergoing sedation-free endoscopy. Investigation for systemic coagulopathies or arterial hypertension should be recommended in the case of non-traumatic orbital hematoma following the exam.

**Summary points**

- Iatrogenic non-traumatic orbital hematoma is a rare complication of upper endoscopy that could compromise the patient's visual prognosis.
- Endoscopists should be aware of this potential uncommon event for a better selection of the patients undergoing sedation-free endoscopy.
- The most likely trigger is an increase in central venous pressure transmitted to the orbital vasculature.
- Treatment options are ranging from observation to needle aspiration and surgery.
- Investigation for systemic coagulopathies or high blood pressure should be recommended in the case of non-traumatic orbital hematoma following upper endoscopy.
